# How does it feel to be helped using minimal cognitive assistance following a traumatic brain injury?

**DOI:** 10.1111/1440-1630.70102

**Published:** 2026-07-09

**Authors:** Mireille Gagnon‐Roy, Nathalie Bier, Frédérique Poncet, Leanne Togher, Mélanie Amaral Dos Santos, Carolina Bottari

**Affiliations:** ^1^ School of Rehabilitation Université de Sherbrooke Sherbrooke (Québec) Canada; ^2^ School of Rehabilitation Université de Montréal Montréal (Québec) Canada; ^3^ Centre de Recherche de l'Institut Universitaire de Gériatrie de Montréal Montréal (Québec) Canada; ^4^ Lethbridge‐Layton‐Mackay Rehabilitation Centre Montréal (Québec) Canada; ^5^ School of Physical and Occupational Therapy McGill University Montreal (Québec) Canada; ^6^ School of Health Sciences University of Sydney Sydney New South Wales Australia; ^7^ Centre for Interdisciplinary Research in Rehabilitation of Greater Montreal Institut Universitaire sur la Réadaptation en Déficience Physique de Montréal du CIUSSS du Centre‐Sud‐de‐l'Île‐de‐Montréal Montréal (Québec) Canada

**Keywords:** activities of daily living, brain injuries, traumatic, caregivers, cognitive assistance, multiple case study

## Abstract

**Introduction:**

Individuals who sustain a moderate or severe traumatic brain injury frequently require cognitive assistance to complete complex everyday activities such as meal preparation. This assistance can be provided by a caregiver or assistive technologies for cognition. Assistance should be provided in an empowering manner, that is, in a way that allows the person to use their residual cognitive abilities. However, little is known about how individuals with traumatic brain injury perceive receiving this minimal assistance. This study aimed to document the perceptions of community‐dwelling adults post traumatic brain injury regarding minimal cognitive assistance provided during a meal preparation task.

**Methods:**

A qualitative descriptive multiple case study was conducted with five individuals having sustained a moderate to severe traumatic brain injury. They participated in three sessions: an evaluation using the IADL Profile, a guided session using a tailored meal preparation task and an interview to explore their perception of minimal cognitive assistance using video recordings. Interviews were analysed using inductive thematic analysis.

**Consumer and Community Involvement:**

Participants with traumatic brain injury were recruited in collaboration with a traumatic brain injury community association.

**Findings:**

Participants expressed general appreciation for the cognitive assistance provided, as it helped them better plan and carry out tasks, thus allowing them to be more efficient, safe and successful. They noted that minimal assistance allowed them time to think and try things on their own and gave them a sense of independence, particularly considering that more explicit assistance was also provided when needed. However, assistance was at times perceived as intrusive or unhelpful, emphasising the need to adjust support based on the individual's emotional response.

**Conclusion:**

Offering minimal cognitive assistance, which allows individuals time to think and make decisions with support, was well perceived by participants. Further studies are needed to personalise cognitive assistance and integrate it into technologies.

## PLAIN LANGUAGE SUMMARY

People who have had a traumatic brain injury may struggle with complex daily tasks like cooking. To help them, caregivers can offer cognitive assistance that guides the person through the activity. This assistance can be minimal, given only when needed, and without telling the person exactly what to do. For example, caregivers can ask questions to help the person think about options, without giving the solution. However, we still know little about how people with traumatic brain injury feel about this help. This study explored how five people with moderate to severe traumatic brain injury experienced receiving minimal cognitive assistance during meal preparation. First, each person was observed doing complex tasks in their home and community. Based on these observations, a meal preparation task was selected for each person. During this task, the person received minimal and personalised help. Afterwards, participants were interviewed. They watched video clips showing moments when they received assistance and talked about how they felt about it. Overall, they appreciated the help. They said it made them feel safer, more efficient, and more successful. They also liked that it gave them time to think and try things on their own. Still, some felt the assistance could sometimes feel intrusive. These findings show that minimal cognitive assistance can be helpful and valued by people with traumatic brain injury. Findings may be useful for caregivers or future technologies. More research is needed to understand how others view this type of support, especially when it comes from technological tools.

**Key Points for Occupational Therapy**

- Minimal cognitive assistance can support performance and empower individuals with traumatic brain injury in complex activities.
- Such assistance recognises their need to do most things on their own while providing enough guidance to experience success.
- Tailored technological assistance could be provided based on their needs.

## 1 INTRODUCTION

Traumatic brain injury (TBI) is one of the leading causes of disability worldwide (Maas et al., 2022). Impairments secondary to TBI are heterogeneous (Chung & Khan, 2013; Covington & Duff, 2021) and chronic (Wilson et al., 2017), as individuals may live for decades with the physical, cognitive, psychological and behavioural impacts of their injury. Due mainly to cognitive impairments, many require assistance for complex everyday activities, even 5 years after their injury (Tate et al., 2020). Executive dysfunction, which affects the ability to formulate goals related to everyday activities, develop plans, execute those plans while multitasking and problem solving and verify whether goals have been adequately attained, is common after a TBI (Godefroy et al., 2010). Performance in complex everyday activities such as meal preparation is especially affected by executive dysfunction due to planning, multitasking and safety requirements (Dubuc et al., 2019; Godbout et al., 2005), resulting in family caregivers having to support or even complete the task for the person. With TBI recovery's dynamic and evolving nature (Corrigan & Hammond, 2013), support for meaningful and complex activities must be tailored and able to transform over time to adapt to the person's evolving needs (Corrigan et al., 2025).

Cognitive assistance can be provided by formal or informal caregivers (whether in the community or in a care facility), through technology or by occupational therapists in the context of cognitive rehabilitation. Cognitive assistance consists of cues, whether verbal, visual, auditory or gestural, aimed at facilitating task performance through support of the person's thinking processes and ensuring safety (Gagnon‐Roy, Bier, Le Dorze, et al., 2024; Le Dorze et al., 2014; Olivares et al., 2016; Van Tassel et al., 2011). Authors have proposed that cognitive assistance be provided in a personalised and progressive manner (Chard et al., 2009; Ownsworth et al., 2017; Seelye et al., 2012; Serna et al., 2010), starting with implicit and nonspecific prompts (e.g., *What could you do?*), followed by a gradual introduction of more explicit assistance, as needed, to ensure success. This form of assistance sits in contrast with cognitive strategies such as errorless learning, which favours the use of explicit cognitive assistance to closely guide the person (Clare & Jones, 2008; Sohlberg et al., 2005). Individuals can thus learn from their mistakes as in error‐based learning (Ownsworth et al., 2017) and internalise metacognitive strategies (e.g., Goal‐Plan‐Do‐Check of the CO‐OP method) (Borujeni et al., 2019). These strategies are integrated by occupational therapists into cognitive rehabilitation processes based on their clinical evaluations (Jeffay et al., 2023). However, the need for assistance is not limited to the window of time in which cognitive rehabilitation is offered. The need for personalised and progressive assistance, provided by formal or informal caregivers, may continue years after injury (Chard et al., 2009; Corrigan & Hammond, 2013; Seelye et al., 2012; Serna et al., 2010). Hence, understanding how this assistance could be provided beyond rehabilitation could be transformative.

Previously explored within the context of a non‐structured IADL evaluation (Gagnon‐Roy et al., 2021; Gagnon‐Roy, Bier, Le Dorze, et al., 2024), minimal cognitive assistance has been proposed as a promising way to support people with TBI in their everyday activities while allowing them to continue to improve. Like the apprenticeship approach described by Ylvisaker (Ylvisaker et al., 2003), minimal cognitive assistance gives individuals time to think and try things out to promote the use of their residual cognitive abilities while providing enough assistance and modelling to guide and support them through complex tasks. Minimal cognitive assistance is provided only when needed—for example, in situations involving safety concerns or triggering strong emotional reactions—or when individuals are unable to progress in their tasks and ask for help despite multiple attempts to encourage their independence. This way of assisting is progressively graded based on responses to previous assistance and previous performances (Gagnon‐Roy et al., 2021; Pelletier et al., 2025). Using personalised and progressive assistance given only when needed, occupational therapists can optimise empowerment in complex everyday activities through the experience of successes while ensuring safety and emotional well‐being.

In addition to being provided by formal and informal caregivers, minimal cognitive assistance can also be delivered through technology. Increasing attention is being given to assistive technologies to aid cognition (ATC) with the thinking that if designed correctly, these technologies may offer the right assistance at the right time, in every instance, every day for years to come. ATCs are particularly promising as they could reduce family caregivers' burden and be complementary to the assistance provided by formal caregivers. To date, ATCs have been found helpful in supporting various everyday activities using personalised assistance, such as during the morning routine (OʼNeill et al., 2018) and meal preparation (Gagnon‐Roy, Bier, Giroux, et al., 2024; Wang et al., 2019; Zarshenas et al., 2023). Using visual and auditory cues, ATCs can guide the person through the steps of the tasks, such as selecting a recipe from a list of customised recipes (Zarshenas et al., 2023) and help them with advanced goal planning (Oyesanya et al., 2021). Certain ATCs have also started to be configured to provide minimal cognitive assistance using activity detection via sensors installed in the person's environment (Olivares et al., 2016; Tekemetieu et al., 2021, 2023), opening the way to the integration of minimal cognitive assistance into the homes of people with TBI.

Nonetheless, little is known about the acceptability of such assistance for concerned individuals. Hence, individuals' responses to minimal cognitive assistance should be explored as this type of assistance requires that the person exert more effort than with explicit assistance. Moreover, before integrating minimal cognitive assistance into ATCs, it is imperative that we first document how the assistance is perceived when provided by a person during complex everyday activities. Essential to independent living, meal preparation was selected for our study considering its high‐level cognitive requirements and regular use in cognitive rehabilitation (Hingst et al., 2024), particularly by occupational therapists (Mohapatra & Kulnik, 2021). The objectives of the study were thus to (1) document the perceptions of people with TBI of the assistance provided by an occupational therapist trained in offering minimal cognitive assistance during complex everyday activities, including meal preparation, and explore the acceptability of such support when provided (2a) by other people (e.g., informal and formal caregivers) and (2b) by ATCs.

## 2 METHODS

A qualitative descriptive multiple case study was conducted (Yin, 2014). This design was selected as it allows for an in‐depth description of a phenomenon (i.e., the perceptions of people with TBI when receiving minimal cognitive assistance) within its context while encompassing diverse individual experiences via the inclusion of multiple cases (Yin, 2014). As the nature and content of minimal cognitive assistance are highly influenced by the context in which it is provided, such as the person who provides the assistance, the supported activities and the observed difficulties (Gagnon‐Roy et al., 2021), documenting context was deemed necessary to understand participants' experience with minimal cognitive assistance. The consolidated criteria for reporting qualitative research (COREQ) (Tong et al., 2007) was used to describe the methodology and results.

This study was approved by the ethical review board of the Centre for Interdisciplinary Research in Rehabilitation of Greater Montreal (CRIR‐1173‐0616), and all participants provided their informed consent.

### 2.1 Positionality

The authors are researchers and students with backgrounds in occupational therapy and speech therapy. The first author (M. G. R.; she/her) was completing her doctoral studies with people with TBI, whereas the third author (F. P.; she/her) was a postdoctoral fellow with over 20 years of clinical experience in brain injury rehabilitation at the time of the study. Both had been trained in the use of minimal cognitive assistance by the senior author (C. B.), an occupational therapist with over 20 years of experience in evaluating people with TBI in their home and community environment and developer of the IADL Profile (Bottari et al., 2010b). M. G. R. was supervised by N. B. and C. B., both professors in occupational therapy. L. T. is a professor in speech therapy, with extensive expertise in TBI research. M. A. D. S. was a research assistant and a master's student in occupational therapy.

### 2.2 Participants

Using a convenience sample, 11 adults with moderate to severe TBI aged 18+ were recruited in collaboration with TBI programs and community organisations in and around Montreal (Canada). Individuals with TBI who had participated in previous studies with our research team (Dubuc et al., 2019; Gagnon‐Roy et al., 2022) were also invited. Participants had to be (a) at least 6 months post‐injury, (b) able to communicate fluently in French or English and (c) interested in resuming or upgrading their participation in meal preparation activities. Participants could be living at home with or without a family caregiver or in a residential facility. Participants also needed to have a confirmed diagnosis, either in their health records or by their membership to TBI community organisations (which only accept individuals who provide a medical file or are referred by a rehabilitation centre following a moderate to severe TBI).

### 2.3 Procedures and data collection

**FIGURE 1:**
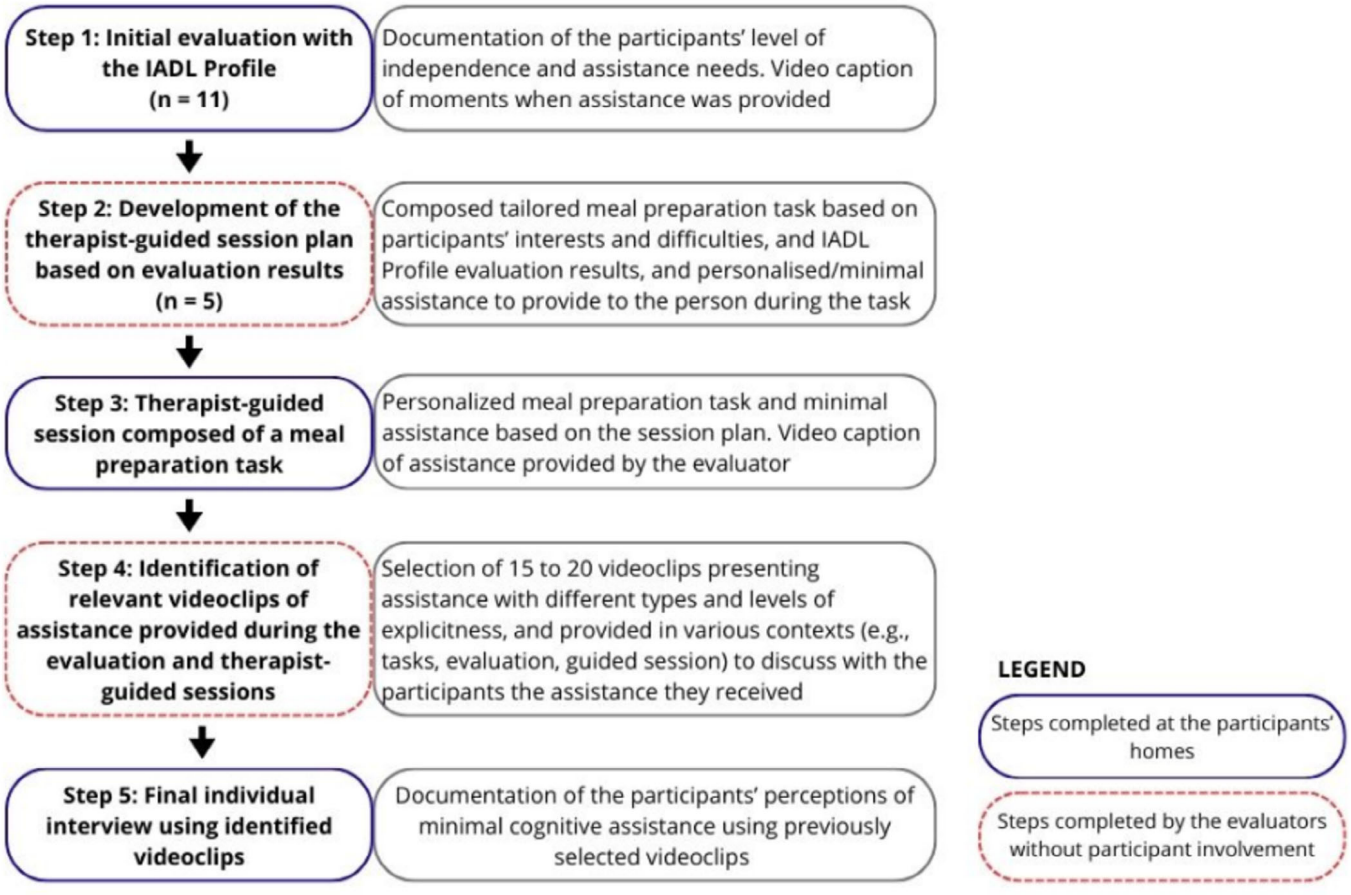
Procedures and steps completed during the study.

The study was conducted in five steps: an initial evaluation (Step 1), the development of a therapist‐guided session plan (Step 2), a therapist‐guided session (Step 3), the identification of relevant videoclips of assistance to guide the discussion (Step 4) and an individual interview (Step 5; see Figure 1). Although all 11 participants took part in the initial evaluation, only participants demonstrating difficulties and requiring assistance during the evaluation (*n* = 5) completed the other four steps and were included in the multiple case analysis. The inclusion of Steps 1 and 3 allowed participants to experience minimal cognitive assistance in a range of situations and activities, first in an evaluation during which assistance was provided in a minimal and exploratory manner to document the person's optimal abilities and second in an individualised meal preparation task during which cognitive assistance was tailored to the participants' needs and difficulties as observed during the evaluation. Individuals' perceptions of the provided assistance were then documented during individual interviews. All steps were completed by two certified occupational therapists (M. G. R. and F. P.) experienced in working with people with TBI. For each participant, an occupational therapist (i.e., M. G. R. or F. P.) was appointed as the main therapist, thus leading the evaluation and guided sessions, while the other videotaped the sessions and took notes. Discussions between the two therapists were conducted and audiotaped after each session and when preparing the guided session plan.

#### 2.3.1 Step 1: Initial evaluation with the IADL profile

Participants were assessed using the IADL Profile, an observation‐based evaluation tool that documents level of independence and assistance needs in complex everyday activities carried out in the home and community environments (Bottari et al., 2009a, 2009b, 2010a, 2010b). Using a minimally structured approach, the IADL Profile assesses the person's ability to formulate goals and plan complex tasks. According to evaluation guidelines, the therapist must provide minimal assistance only when needed to ensure the client's safety and ability to progress in the task. By doing so, the therapist can assess the person's independence and document the extent to which the person is able to think through and carry out the task. This evaluation approach also allows therapists to explore the usefulness of various types of assistance (Gagnon‐Roy, Bier, Le Dorze, et al., 2024; Gagnon‐Roy et al., 2021) and use evaluation results to guide subsequent treatment interventions.

The IADL Profile consists of eight tasks divided into three scenarios: preparing a hot meal for guests (e.g., going to the grocery store, grocery shopping and preparing a meal), budgeting and obtaining information about bus schedules for an upcoming trip to a neighbouring city. Grocery shopping (including going to the grocery store) was selected as a functional outcome (Cole et al., 2025), considering its link with meal preparation. The two other tasks were also assessed considering their complexity and association with meal preparation, as the person must be able to find relevant information (e.g., a recipe) and budget cooking expenses.

Each task is scored on four task‐related operations using a 5‐point ordinal rating scale: independent without difficulty (4), independent with difficulty (3), requires verbal or physical assistance (2), requires verbal and physical assistance and dependent (0), totalling a maximum possible score of 116 points. Based on factorial validation studies suggesting six main factors (Bottari et al., 2009b), an average score is then calculated to obtain task (e.g., preparing a meal) or combined tasks (e.g., going to the grocery store and grocery shopping) scores, which are interpreted using the same scale. Three ecological indexes related to executive functioning (complex planning, task execution and action working memory) can also be calculated by averaging specific task‐related operations across observed tasks (Bottari et al., 2009b). Following the evaluations, each therapist independently scored the IADL Profile. They then compared their scores and discussed their understanding of the participants' assistance needs.

#### 2.3.2 Step 2: Development of the therapist‐guided session plan based on evaluation results

The therapists then met to develop personalised session plans for each participant. Plans consisted of a tailored meal preparation task and a personalised session goal (e.g., to prepare a meal within an adequate time) based on participants' interests, difficulties, and IADL Profile evaluation results, including level of independence and assistance needs. Personalised cognitive assistance to guide the person with the minimal or least amount of assistance needed (e.g., read the instructions with the person and ensure that all ingredients and tools are ready before starting the recipe) and, in case of specific difficulties, was also defined based on observations made during the evaluation and cognitive rehabilitation principles (Dawson et al., 2013; Jeffay et al., 2023). Types of assistance, as well as their progression from minimal‐implicit to explicit depending on the person's actions and documented difficulties, were selected based on previous work on minimal cognitive assistance (Gagnon‐Roy, Bier, Le Dorze, et al., 2024; Le Dorze et al., 2014). Because the objective of the session was to guide the person in completing a meal preparation task in an acceptable time and manner, predefined assistance could be more explicit than what had initially been provided during the evaluation session.

#### 2.3.3 Step 3: Therapist‐guided session composed of a meal preparation task

The therapist‐guided session was then completed at the participant's home. To ensure that participants were able to complete the selected task (e.g., prepare a dessert using a new recipe), the duration of the session was not determined in advance, although the estimated preparation time for the recipes was less than an hour. Personalised and minimal cognitive assistance was provided throughout the session to successfully attain the goal, based on the assistance that was predefined in the session plan (Step 2) and observed difficulties experienced by the participants.

#### 2.3.4 Steps 4 and 5: Videoclip selection and final individual interview

In Step 4, between 15 and 20 videoclips illustrating moments of assistance that occurred over the evaluation and therapist‐guided sessions were selected for each participant. This was done in preparation for a 60‐min individual interview with the participants to discuss specific moments when minimal cognitive assistance was provided by the therapist to help participants progress in the task (Step 5).

Videos from both sessions were carefully viewed and coded by the first author (M. G. R.) to (a) identify relevant moments and (b) describe the types of assistance (Gagnon‐Roy, Bier, Le Dorze, et al., 2024) that were provided and their context (e.g., task, operations). Moments of assistance selected for discussion were not limited to the meal preparation task but could include any IADL Profile task to ensure diversity regarding the type of assistance provided, its context and the difficulties it aimed to support. During the interview, videoclips were presented to the participant. After the viewing of each videoclip, participants were asked to express their thoughts about their performance (*What do you think about your performance at that moment?*), the assistance they received (*How did you find our help? What types of assistance did you prefer? Disliked?*) and whether other types of assistance could have better helped them progress in the task. Following the viewing of selected videos, two supplementary questions were asked to explore the acceptability of minimal cognitive assistance beyond the context of the study to imagine this same type of assistance being offered (a) by other persons, such as informal or formal caregivers (*Would you like to receive assistance from a caregiver?*) and (b) via ATCs (*Would you like to receive assistance from a technology‐based solution?*). Interviews and viewing of the corresponding videos were conducted by the first author (M. G. R.) at the participants' homes.

### 2.4 Data analysis

#### 2.4.1 Case description

Case descriptions included data from the IADL Profile score sheet, video data from the evaluation and therapist‐guided sessions (e.g., types of assistance and context in which it was provided), the guided session plan and transcripts of the discussions between the occupational therapists. These data were analysed and triangulated, i.e., combined using a matrix and compared to obtain a more comprehensive and credible understanding of each case, to identify the participants' main difficulties and assistance needs. Each case was first prepared by the first author (M. G. R.) and validated by the second therapist (F. P.).

#### 2.4.2 Qualitative analysis

Following case descriptions, final interviews were transcribed and individually analysed using inductive thematic analysis based on the method described by Miles et al. (2019). Transcripts were carefully read through by the first and fourth authors (M. G. R. and M. A. D. S.) to identify codes describing the perceptions of participants regarding minimal cognitive assistance. Two transcripts (40%) were first coded by one of the authors (M. G. R. or M. A. D. S.) and reviewed by a second, whereas the remaining were coded by M. G. R. Following initial coding, preliminary themes were identified for each case to capture the participants' unique and individual experiences when receiving minimal cognitive assistance. To ensure methodological rigour, an audit trail of the coding process, including reflective notes, was kept and reviewed multiple times across the data analysis process.

A cross‐case comparison analysis was conducted. Coding lists from the five cases were merged into one while ensuring that codes specific to each case were maintained. This allowed us to merge similar codes between cases, thus capturing similar perceptions across participants, while highlighting the specific individual experiences. Only well‐described and saturated codes were used. The final coding list was used to regroup codes into themes, all of which were validated by the last author (C. B.). Finally, we built a matrix to display the main themes and related codes for each participant, allowing comparison between cases (see Table 3).

## 3 FINDINGS

Five adults with a moderate to severe TBI, at least 10 years post‐TBI, were included in the multiple case study. Their meal preparation habits varied considerably, with Anna having all her meals prepared for her, three participants (James, Mary and Sarah) having previously taken part in collective cooking activities in a community TBI program and Brian living alone, thus preparing meals on a regular basis. Participants' characteristics and evaluation scores are presented in Table 1. Observations from the IADL Profile, including the selected recipe, the main difficulties and the minimal assistance provided by the therapist, are presented in Table 2. Selected moments of assistance for the individual interviews are described in the Supporting Information (see Description of the selected moments of assistance presented during the individual interviews).

**TABLE 1:** Characteristics and functional assessment of participants*.*

	James	Mary	Anna	Sarah	Brian
Characteristics					
Age (years)	34	37	48	42	61
Time since TBI (years)	11	18	24	30	11
TBI severity	Severe	Severe	Severe	Moderate	Moderate
Occupation (post‐traumatic brain injury)	Unemployed	Unemployed	Unemployed	Unemployed	Unemployed
Driving	Yes (scooter)	No	No	No	Yes
Living situation	With brother	With boyfriend	Residential facility	With parents	Alone
Motor impairments	Difficulty walking (limp) due to spasticity in left leg	None reported	Manual wheelchair user, hemiplegic, tremors in upper limbs	None reported	None reported
Therapist	F. P.	M. G. R.	M. G. R.	F. P.	F. P.
IADL Profile^a^					
Grocery shopping/going to the grocery store (/4)	3.25	2.50	0.00	2.13	3.25
Preparing a hot meal for guests (/4)	3.25	2.50	0.50	2.25	2.50
Eating with guests and cleaning up after (/4)	2.14	3.71	1.29	3.14	4.00
Putting on outdoor clothes (/4)	2.75	4.00	0.00	4.00	4.00
Obtaining information (/4)	2.00	2.33	0.00	0.00	0.00
Making a budget (/4)	1.33	0.67	0.00	0.00	2.00
Complex planning (/4)^b^	2.86	2.43	0.00	1.57	2.00
Task execution (/4)^b^	2.50	2.50	0.25	1.50	2.88
Action working memory (/4)^b^	2.71	2.07	0.00	1.07	2.00
Total (/116)	75	81	11	64	86

^a^ A higher score is associated with a higher level of independence: 4 corresponds to independent without difficulty; 3 corresponds to independent with difficulty; 2 corresponds to requires verbal assistance; 1 corresponds to requires both physical and verbal assistance; and 0 corresponds to dependent (Bottari et al., 2009b).

^b^ Three ecological indexes identified in factorial validation studies (Bottari et al., 2009b).

**TABLE 2:** Main difficulties and minimal cognitive assistance provided to participants during the IADL Profile evaluation*.*

	Selected meal	Main experienced difficulties	Minimal cognitive assistance provided by the therapist and examples of selected moments of assistance
James	Ground beef with onions accompanied by hummus and pita bread (familiar meal prepared without a recipe)	*Planning*: Difficulty adjusting his plan according to the evaluation context and his own requirements (e.g., having to buy halal meat, balance impediment). Difficulty identifying expenses and allocating amounts in conformity with the context provided in the budgeting task *Carrying out:* Difficulty initiating tasks outside his habits (e.g., setting the table, cleaning up after) Difficulty searching and finding the right information on the internet *Verifying the attainment of the goal:* Slightly burned his onions Unable to complete the budgeting task despite explicit assistance	*During the grocery shopping and meal preparation tasks:* Clarifications and implicit assistance to stimulate thought processes and question him (i.e., challenging) to help him adapt his plan according to his abilities and context. Example: Clarification to help James understand the context and formulate the goal of grocery shopping: *‘We would like to see you function outside of the kitchen. You proposed a meal with what you had in your fridge. What would you do if you had nothing?*’ *During the tasks of obtaining information and budgeting:* Increasingly explicit assistance, such as stimulating and restarting thought processes, challenging and cues. Example: Assistance questioning James to help him think about the expenses he forgot: ‘*You plan on putting $13,080 (of the $25,000 annual amount) on the car?*’
Mary	Meatloaf with a pasta side dish and homemade applesauce (selected by Mary before the evaluation)	*Planning*: Despite being able to identify some strategies by herself (e.g. read the recipe and make a grocery list before going to the grocery store) and explain its main steps, difficulty initiating them and adequately planning her meal *Carrying out:* Easily frustrated and anxious when confronted with difficulties, such as when searching a recipe on the internet Difficulty monitoring her performance and her meal Difficulty multitasking Difficulty calculating and allocating adequate amounts of money during the budgeting task *Verifying the attainment of the goal:* Did not buy enough ingredients for her recipe Initially refused to do the budgeting task	*During the grocery shopping and meal preparation tasks:* Multiple interactions to encourage her independence and motivate her to do as much as possible by herself. Progressive explicit assistance to question, cue and remind Mary of the strategies she had already proposed so that she could initiate them, as well as reactivate her own knowledge. When becoming frustrated, explicit step‐by‐step assistance (i.e., cues) to de‐escalate her behavioural response to the situation and guide her through problem solving. Example: Interaction aimed at encouraging independence: ‘*You organise yourself like you want to*’. Example: Assistance to question Mary (i.e., challenging) as she begins to prepare the pasta before placing her meatloaf in the oven: ‘*Now, just to make sure, what are we doing?*’ *During the tasks of obtaining information and budgeting:* Progressive assistance to encourage her to do as much as she could by herself and cue her through the elements of the task that she was unable to do on her own.
Anna	Pasta with frozen vegetables and soya sauce (simple new meal without following a recipe, prepared at her boyfriend's apartment with an adapted stove)	*Planning*: Difficulty and slowness in selecting an appropriate meal and detailing the steps to complete *Carrying out:* Difficulty finding the items she needs Requires assistance to move around the cauldrons on the stove Difficulty following the steps and monitoring her cooking *Verifying the attainment of the goal:* Inability to go to the grocery store due to severe physical impairments Difficulty adapting her meal despite limited quantities and too much seasoning Inability to complete both the obtaining information and budgeting tasks due to fatigue and need for extensive assistance	*During the grocery shopping and meal preparation tasks:* Implicit assistance to stimulate her thought processes and help her propose potential solutions Progressively explicit cues to help Anna detail her ideas, select the best one and apply it to the meal preparation task. Physical assistance to assist with cauldrons and finding items outside her reach. Example: Cues to follow the steps so that the pasta and vegetables are well‐cooked: ‘*How much time is required to cook the vegetables? If we just add the pasta to the water and that takes approximately the same amount of time, what are we doing with the vegetables?*’ *During the tasks of obtaining information and budgeting:* Explicit step‐by‐step assistance not sufficient; both tasks were incomplete due to fatigue. Example: Assistance to stimulate thought processes and help her find alternatives: ‘*Are there other places you could find this info? If you were at your home?*’
Sarah	Zucchini and tomato pasta (new recipe selected by Sarah and her mother before the evaluation and doubled due to the number of persons present)	*Planning*: Difficulty identifying the ingredients she needs Difficulty identifying alternatives and modifying her plan accordingly *Carrying out:* Difficulty making a decision by herself Difficulty following a recipe. The step order was not optimal (e.g., cuts all ingredients before boiling water) and particularly slow (2 h for a 45‐min recipe) Difficulty identifying her mistakes and correcting them *Verifying the attainment of the goal:* Inability to complete the budgeting task due to fatigue and need for extensive assistance	*During the grocery shopping and meal preparation tasks:* Implicit assistance to stimulate thought processes and question the next steps and quantities to help Sarah follow the recipe and be efficient. Explicit cues and physical assistance to be more efficient (e.g., to move cauldrons and help with cutting) Example: Cue to help Sarah be more efficient when following the steps: ‘*You could do that later, and maybe begin boiling water. Because it will take some time to cut all your vegetables*’. *During the tasks of obtaining information and budgeting:* Implicit assistance aiming to stimulate thought processes and challenge Sarah, before cueing her in the next steps Step‐by‐step assistance not sufficient for the budgeting task due to fatigue. Example: Assistance to challenge Sarah about the objective of the task: ‘*What was the mode of transportation I mentioned?*’
Brian	Omelette with a vegetable soup and pre‐made muffins (familiar meal in line with his dietary restrictions, including diabetes, celiac disease)	*Planning*: Difficulty finding meal alternatives outside of his habits (i.e., for more than one person) and adjusting his plan accordingly (took more than 20 min to plan his meal and initiate going to the grocery store) *Carrying out:* Difficulty initiating the meal preparation and grocery shopping tasks Slowness when preparing his meal Difficulty proposing expenses and related amounts other than those specific to his income and living expenses in the budgeting task Requires step‐by‐step assistance to obtain information and use the internet Difficulty with abstraction and problem solving	*During the grocery shopping and meal preparation tasks:* Implicit assistance to reactivate and stimulate Brian's thought processes, followed by explicit assistance such as cues to help him choose between multiple ideas. Explicit assistance to initiate the task despite the time (before noon). Example: Assistance to restart Brian's thought processes to help him select a meal: ‘*Finally, what will you do? You have a lot of ideas*’. *During the tasks of obtaining information and budgeting:* Explicit step‐by‐step assistance to use the internet to obtain information Example: Assistance to challenge and think about alternate ways of obtaining information: ‘*Do you think there could be another way to obtain this information besides calling or going in person?*’

### 3.1 James

During the IADL Profile, James was independent with difficulty for going to the grocery store/grocery shopping and for the meal preparation task, despite slightly burning his onions and requiring assistance to adjust his plan to the evaluation context. Considering his performance when cooking a familiar recipe and his interest in cooking more complex ones, the goal of the guided session was to prepare a lasagna with minimal cognitive assistance, aligned with his aspiration to be able to cook one. Also, as most of his meals were prepared by his family, the session was presented as a leisure activity, taking place in a nonfamiliar environment, that is, a community kitchen of the community TBI association. As James's main difficulties were in planning, the therapist decided to initially present him with the recipe. According to the session plan, minimal cognitive assistance was then provided progressively to stimulate his thought processes (*How are you going to do it?*) and, if not initiated automatically by James, cue him to review the ingredients he needed before beginning the meal preparation task. In case of difficulties, initial assistance such as questioning (e.g., *What are you doing right now?*) and stimulating thought processes (e.g.*, Where could you look to verify this information?*) were considered, followed by increasingly explicit assistance such as cues (e.g., *Have you cooked the pasta?*).

Overall, the lasagna preparation took 3 h to complete. Multiple times, James had difficulty engaging in the task and initiating the next steps, which required that minimal cognitive assistance be given and quickly progressed by the therapist. Implicit assistance, such as restarting thought processes and questioning (e.g.*, Are you sure? What could you do during the time water is boiling?*), was first provided, followed by increasingly explicit cueing (e.g., *You need to cut your onions smaller*) to keep James engaged in the task. He had difficulty structuring the task and following through. This difficulty was exacerbated whenever James had to complete several steps simultaneously, such as shredding cheese and cooking pasta. Although the task was difficult for the participant, he was able to complete it with help from the therapist and was proud of his accomplishment.

### 3.2 Mary

During the IADL Profile evaluation, Mary required verbal assistance for both going to the grocery store/grocery shopping tasks and the meal preparation task. She was taking antidepressants and used marijuana regularly to reduce anxiety, and frequently demonstrated signs of anxiety and lack of confidence during the evaluation. Considering her performance, she was asked to select and prepare a new dessert recipe. This task was selected as it involved finding a recipe on the web and could be replicated by Mary at home. When presented with the session goal, she chose to use a cake mix and add homemade creaming. Before beginning the task, the therapist reviewed with her the main difficulties and strengths that had been observed during the evaluation to help her anticipate potential difficulties and improve her self‐confidence. Based on observed planning difficulties during the evaluation, minimal cognitive assistance was then provided to stimulate Mary's thought processes and encourage her to review the steps and make sure she had all necessary ingredients. In case of difficulties, other assistance to stimulate her thought processes (e.g., *How could you do that?*) and question her (e.g.*, How long do you think it will take?*) were identified, followed by more explicit assistance such as cues.

Overall, the therapist‐guided session (including the recipe selection) took around 80 min. Mary mainly received minimal cognitive assistance to detail her plan and follow through efficiently and successfully. As previously defined in the session plan, assistance was mostly aimed at stimulating her thought processes (e.g., *How will you organise yourself? What will you do first?*), questioning what she is doing (e.g., *Before preparing the icing, how will we know the cakes are ready?*) and cueing her when previous more implicit assistance was not successful (e.g., *You can stop mixing the butter before it turns into cheese*). Although she was anxious, which could have been exacerbated by the camera's presence, Mary was proud of the result and her ability to cook ‘by herself’.

### 3.3 Anna

The IADL Profile evaluation was conducted in Anna's boyfriend's kitchen, as it provided access to an adapted stove, because her boyfriend was also a wheelchair user. In addition to being unable to go to the grocery store due to the distance to the closest grocery store (around a 10‐min walk) and her physical impairments, she was found to be dependent for the meal preparation task, despite receiving both verbal and physical assistance to help her plan and prepare her meal. Given Anna's severe difficulties and her objective to prepare simple meals on her own using a small oven, a vegetable quiche recipe was selected and adapted for the guided session. All required ingredients were brought to her room. Considering Anna's difficulties when planning and carrying out tasks, the therapist first reviewed with her the main difficulties that had been observed during the evaluation and reviewed the steps to prepare her meal. As Anna required many cues during the evaluation, the therapist planned on quickly progressing the assistance to more explicit cues to foster success. Minimal cognitive assistance thus consisted of a few implicit assistances to stimulate her thought processes, followed by increasingly explicit cues to guide the next steps (e.g., *What is the next step in the recipe?*). Using this minimal and personalised approach, the therapist encouraged Anna to think about her plan (*Can you explain what you are planning to do to prepare your recipe?)* and to take out all the ingredients and tools she would need prior to starting.

Overall, the session took around 3 h. Anna required step‐by‐step assistance, first by stimulating thought processes with implicit assistance (*Now, what are we doing?),* then by questioning (*What should you do before? Did you check the ingredients?*) and finally using explicit cues. Minimal cognitive assistance had to be quickly progressed and explicitly provided, as she required mostly cues and action priming to progress in the task. In addition, physical assistance was provided to stabilise dishes, move items around and monitor cooking. Ultimately, Anna was found to require extensive assistance throughout the guided session, although she was happy to have prepared a meal.

### 3.4 Sarah

During the IADL Profile evaluation, Sarah required verbal assistance for both the going to the grocery store/grocery shopping tasks and the meal preparation task. Considering Sarah's lack of efficiency during the evaluation, she was asked to select and prepare a new recipe in a limited time. She selected a couscous recipe with vegetables and feta cheese. To optimise Sarah's efficiency, minimal cognitive assistance was provided to first stimulate her thought processes and help her find ways of being more efficient (*What would your strategy be to be more efficient?*). When not initiated automatically by Sarah, more explicit assistance was provided to encourage her in completing relevant preparation steps, such as reading through the steps and taking out the ingredients and tools she would need. Minimal cognitive assistance also aimed to restart the task in case of delays between two steps (*What is the next step?*) and stimulate thinking about efficiency (*What could you do in the meantime?*), before progressing to more explicit assistance to help Sarah be more efficient.

Overall, the meal preparation task took 30 min for a 15‐min recipe. Sarah required assistance to organise steps efficiently (e.g., *Now that you selected your recipe, what will you do?*), make sure to follow through the recipe and reduce delays associated with transitions. Minimal cognitive assistance was provided progressively to help her think about the next steps (*What could you do next?*), initiate them (*Go!*) and finally cue her when difficulty with decision‐making and lack of cooking knowledge was observed. Ultimately, Sarah was happy with the results and her efficiency.

### 3.5 Brian

Despite having to regularly prepare meals, Brian required verbal assistance for the meal preparation task. Considering Brian's difficulties and desire to prepare new recipes on his own, he was asked to find a new recipe on the internet, adjust it for six persons and cook it. Before the guided session, a website, including simple recipes, was identified as a suggestion for Brian. Planned minimal cognitive assistance consisted of initially providing implicit assistance to stimulate thought processes (e.g.*, Where could you search for a new recipe?*), scaffold on previous interactions (*Last time, you talked about the Internet)* and recall memories (*Have you ever followed recipes on the Internet?*), followed by progressively explicit assistance such as cues.

Overall, the intervention took 3 h, including recipe selection, grocery shopping and cooking. Planning and recipe selection (spaghetti) were difficult, requiring minimal cognitive assistance progressing from implicit assistance to stimulate his thought processes (*How could you find another idea of meals?*), to explicit cues to guide him through the internet search (*You could write the recipe [as keyword]*). He was also reminded of the difficulties he experienced during the evaluation to help him question himself about his planning and consequently adjust the recipe for six persons (*Do you remember what happened with the quantities last time? I am not sure you have enough water*). Moreover, despite providing minimal assistance, explicit assistance such as cues and physical assistance was required to optimise efficiency and success (*Shouldn't they be cut? Would it be okay if I cut them in half to ensure the pasta cooks properly*). Ultimately, Brian was able to select and cook a new recipe, although he required continuing assistance.

### 3.6 Perceptions of people with TBI regarding minimal cognitive assistance

Five themes described the perceptions of participants regarding minimal cognitive assistance provided during the evaluation and guided sessions: (1) general appreciation of minimal cognitive assistance; (2) feelings of independence when provided with time and space to think; (3) perceived personalised support to help plan complex tasks; (4) perceived helpfulness of minimal cognitive assistance in being organised, efficient and safe; and (5) perceived role of minimal cognitive assistance in improving emotional well‐being during the realisation of challenging tasks. Themes and associated codes are presented in Table 3. Participants' reflections on the expected acceptability of minimal cognitive assistance when provided by caregivers or ATCs are described in the last section.

**TABLE 3:** Themes and codes describing the perceptions of participants regarding the minimal assistance provided during the evaluation and guided sessions.

Themes and definition	Included codes	James	Mary	Anna	Sarah	Brian
General appreciation of minimal cognitive assistance *Participants generally appreciated minimal cognitive assistance, especially when provided by a calm and knowledgeable person. However, assistance was sometimes found to be incomprehensible or too abstract, not sufficiently directive or even pointless*	General appreciation of the minimal cognitive assistance provided during the evaluation and intervention	x	x	x	x	x
	Confidence in the therapists' assistance considering their level of knowledge regarding meal preparation	x	x			
	Better appreciation when minimal cognitive assistance is provided by a calm person, as it reduces stress		x			
	Lack of understanding of some assistance objectives and content	x				x
	Inability to progress in the task despite minimal cognitive assistance, which may be too abstract (e.g., hypothetical situations) or implicit (questions)	x				x
	Perception that some assistance, such as reminders and demos, were not necessary as they already knew the info			x		x
Feelings of independence when provided with time and space to think *Through guidance and mostly implicit assistance (*e.g.*, questions), participants felt they could do the task mostly by themselves, as they had time, space and autonomy to think and find solutions. More directive assistance was however needed to maintain successes and a positive experience*.	Support from minimal cognitive assistance in completing and managing the task mostly by themselves made them feel independent	x	x			
	Space, time and autonomy to think by themselves after receiving minimal cognitive assistance	x	x	x	x	
	Perceived need and appreciation of more directive and explicit assistance in more challenging situations		x	x	x	
	Perceived need for more directive assistance after having the time to think about potential solutions			x	x	
	Skills acquisition through demonstrations of cooking techniques	x			x	
	Demonstration of specific actions to optimise task efficiency and success	x	x	x	x	x
Perceived personalised support to help plan complex tasks *Participants appreciated the minimal cognitive assistance to help them plan and ensure they had everything before beginning complex tasks*.	Preparation time and verification of ingredients and cooking tools before beginning the meal preparation task		x	x	x	
	Going through recipe steps to question how the task should be planned and organised	x		x	x	
	Minimal cognitive assistance aiming to stimulate thought processes and suggestions to help identify alternatives to meals					x
	Reminders of previous ideas to help in selecting one				X	
Perceived helpfulness of minimal cognitive assistance in being organised, efficient and safe during meal preparation *Participants appreciated the minimal cognitive assistance provided when carrying out the tasks, as it helped them be more organised, efficient and aware of their mistakes*.	Suggesting taking breaks during the task to evaluate the situation and correct themselves		x	x		
	Challenging and questioning to reevaluate a situation and identify their mistakes	x	x		x	x
	Encouragements to stay engaged and continue the task or start a new task during a waiting time (e.g., cooking time)	x			x	x
	Continuous minimal cognitive assistance to follow through the steps in an efficient and successful way	x	x		x	
	Reminders to keep in mind previous mistakes and next steps and make sure not to forget anything		x	x	x	x
Perceived role of minimal cognitive assistance in improving emotional well‐being during the realisation of challenging tasks *Despite having frustrations and feelings of intrusion and stress, participants also expressed how minimal cognitive assistance could help them reduce their stress and improve their confidence in their abilities*.	Frustrations and feelings of intrusion in response to assistance received		x	x		
	Stress associated with the evaluation and intervention contexts and unfamiliar environment	x	x	x	x	
	Reduction of stress and anxiety secondary to guidance received and observed progress in the task		x		x	
	Improvement of self‐confidence and pride in their own abilities	x	x			

#### 3.6.1 General appreciation of minimal cognitive assistance

All participants mentioned globally appreciating the assistance they were provided to complete the sessions. For example, Sarah felt that she would benefit from receiving minimal and progressive assistance to help her in some daily activities: ‘*Like what [the therapist] was doing, maybe I'd need it more often, because it was stuff [about cooking] I did not remember*’. Although he usually prepared meals alone, Brian also enjoyed having assistance in the task: ‘*Three‐quarters of the time, I'm on my own […]. But at least I had someone there. [The therapist] was telling me the steps to take before doing this. […] She was a great help*’. As mentioned by James and Mary, the fact that the evaluator was calm and familiar with cooking played a part in how they perceived the assistance: ‘*When you know that she knows what she's doing, it helps you trust her*’. (James).

Participants, however, highlighted situations when minimal cognitive assistance was not helpful. Brian explained that ‘*[the therapist] asked [him] questions, and sometimes [he] realized he didn't know what she's talking about…*’. In different situations, the assistance was not sufficient to help Brian find alternatives and choose one, thus highlighting the need for more explicit assistance. Furthermore, some assistances were perceived as unnecessary as they aimed to remind participants of elements they already knew, such as when cooking techniques were demonstrated to Anna although she already practised them in cooking classes. As a result, although participants globally appreciated the assistance they were given, some assistance could have been modified to optimise guidance during meal preparation, such as by providing more explicit cues.

#### 3.6.2 Feelings of independence when provided with time and space to think

Most participants appreciated how minimal cognitive assistance was provided in a progressive manner, allowing them time and space to think about solutions and select one. By being given time, but also more assistance when needed, participants felt they were able to do most things by themselves, even feeling that they had completed the tasks independently, as illustrated by Mary: ‘*It went well. I needed some help, but basically, I managed on my own*’. By being offered implicit assistance such as questions to stimulate thought processes, participants highlighted how it gave them time and space to really try by themselves and develop their own abilities: ‘*It gave me time to understand, to know*’. (Sarah). This was especially important for Anna who mentioned: ‘*I wanted to find it by myself*’.

At some point, all participants were provided explicit assistance to progress in the tasks and attain their objectives. This level of assistance was especially appreciated in challenging situations (e.g., budgeting and obtaining information), when participants were overloaded with information or when they lacked the knowledge and skills to progress. For example, Mary explained: ‘*[Directive assistance was] good, because I didn't know how to do it, the melted butter. I really didn't know how to do it*’. Participants felt that, because they already had the time and space to think by themselves, more directive assistance was needed to be able to progress in the task: ‘*I think I needed it, since I hadn't already found the information by myself* (Sarah)’. Anna further mentioned that after some time of trying by herself (e.g., 15 min), the assistance provided to her was necessary.

Although only Anna had physical impairments requiring physical assistance, all participants required some demonstrations (i.e., showing how to do a specific action) from the therapist. Although such assistance was not necessary per se to attain their objectives, they appreciated it as it helped them be more efficient and obtain a better result. For example, Sarah explained that the help from the therapist to pour the contents of the cauldron (which was exceptionally heavy due to the preparation of a double recipe) improved her already slow performance: ‘*With the quantity in the cauldron, it would have taken me a little longer to manage on my own than if I hadn't had anyone to help me hold it*’.

#### 3.6.3 Perceived personalised support to help plan complex tasks

As planning is frequently difficult after a TBI, participants emphasised that they had received—and appreciated—minimal cognitive assistance to support planning before starting their tasks. For some participants (James, Anna and Sarah), this assistance consisted of being given some time before the task to go through each step and question themselves about how they would complete it. Sarah expressed that this process allowed her to really understand the task requirements: ‘*Maybe it made me realise several things. […] Because before she told me, I didn't seem too sure of all the steps I had to think about*’. Anna especially liked this process, which helped her: ‘*I was revising. The more I think, the more I [know] what I'm doing*’. Mary, Anna and Sarah were also invited to verify if they had all the ingredients and tools they needed and to take them out before starting the task. Such assistance was found helpful to reduce unnecessary movements around the kitchen and to be prepared.

Because of his difficulty finding alternatives outside of his own habits, multiple moments of assistance aiming to stimulate thought processes, as well as suggestions and potential ideas, were provided to Brian. Although it remained difficult, he found it helpful: ‘*She gave me ideas. […] Well, look at that [recipe], maybe it won't be that bad. Then it was from there that I… I'll do this one*’. (Brian). When confronted with a situation during which she had difficulty selecting an option, Sarah highlighted the extent to which she appreciated the minimal cognitive assistance that aimed to remind her of previous ideas she had had (i.e., scaffolding). This helped her compare information and select the best option: ‘*Sometimes it's hard for me to think of everything at the right time*’.

#### 3.6.4 Perceived helpfulness of minimal cognitive assistance in being organised, efficient and safe

Participants also praised the assistance provided to support them when carrying out complex tasks. First, they highlighted how minimal cognitive assistance helped them identify their mistakes and correct themselves, whether by taking a break, through implicit assistance such as questioning or with progressively explicit cues. James explained that questioning allowed him to ‘*dot the i's and cross the t's. Because there are several things that you don't take into consideration […] but then, [with questioning] you look at it from another point of view*’. Consequently, minimal cognitive assistance helped him reevaluate the situation and find a solution. Reminders were also found relevant in carrying out the task, as they could help the participants consider previous mistakes and future steps and make sure not to forget anything. For example, Brian explained that having been reminded that he had not made enough food during the evaluation, he was able to adjust the quantities during the guided session. Sarah also appreciated when the therapist reminded her to add her onions: ‘*I don't know how long it would have taken me to realise it. I seemed to have forgotten them again*’.

Assistance aiming to optimise performance was also appreciated by most participants. James especially praised this assistance as he mentioned: ‘*When you're doing any task, you don't want to drag yourself through it […] you want it to be enjoyable to cook*’. Because meal preparation tasks took more time than initially expected by participants, they all appreciated how minimal cognitive assistance helped them stay organised and follow through the recipe: ‘*I think it helped me realise more quickly where I was at* (Sarah)’. Participants confirmed that more directive and explicit assistance was needed to complete complex tasks within an acceptable timeframe.

#### 3.6.5 Perceived role of minimal cognitive assistance in improving emotional well‐being during the realisation of challenging tasks

Finally, most participants mentioned both negative and positive emotional impacts associated with the evaluation and therapist‐guided sessions. These included not only stress, feelings of intrusion and frustrations but also pride at having managed to do the task with help. James expressed: ‘*When someone's standing next to you and telling you what to do… You have doubts about the procedure…*’. He further explained: ‘*[the therapist] was there, not like a peg, but like “are you sure?*” *and then I did not even know [how] to take it*’. Mary also highlighted a situation during which she felt frustrated, as she could not find the recipe she wanted.

Nonetheless, despite these somewhat negative emotions, participants highlighted how minimal cognitive assistance helped them regulate their stress while navigating through complex tasks, ultimately feeling proud and more confident about their own abilities. Although she was initially frustrated to have to do some tasks, Mary ‘*was happy to have done it. [She] did it, so [she] was happy*’.

### 3.7 Potential acceptability of minimal cognitive assistance when provided by family members or ATCs

Most participants expressed that minimal cognitive assistance could be provided by a family member or a staff member (in the case of Anna). For example, Mary explained that the assistance already provided by her boyfriend (usually explicit and directive) was appreciated and helped her complete tasks at her home. Sarah also highlighted how minimal cognitive assistance could be motivating if well provided: ‘*If [my parents] could watch me like that. And tell me when I'm out of order, when I'm forgetting something. That's something I'd need*’. However, for Brian, having someone that provided him assistance was not acceptable as he was living alone and trying to do most things by himself: ‘*I try to do things myself. If I keep asking someone for help, I'll never get around to doing my own thing*’.

Finally, participants appreciated the idea of using an ATC that could provide them with minimal cognitive assistance. Four participants (James, Mary, Sarah and Brian) highlighted that such assistance could help them follow a recipe and ‘*find [where they are] when [they] skip a step*’. (Sarah), including by having the possibility to check steps when completed. However, the need for training and assistance with technologies was identified, as well as some doubts about the assistance ATCs could provide: ‘*[Technology can] help you structure steps, but can it be as efficient as someone helping you?*’ (James). Nonetheless, despite some doubt, James felt that technologies are relevant and would likely have the potential to help him be more efficient in complex activities such as meal preparation.

## 4 DISCUSSION

Using a qualitative descriptive multiple case study, this study aimed to document the perceptions of five individuals with TBI when receiving minimal cognitive assistance from an occupational therapist during complex everyday activities such as meal preparation. Additionally, it explored the potential acceptability of such assistance if provided by informal and formal caregivers or using ATCs. Overall, participants appreciated having the time and place to problem solve, while receiving enough assistance to progress in the task. Although assistance was not always perceived as helpful or even necessary, they felt that minimal cognitive assistance—provided in a progressive and personalised approach—guided them through the planning step and helped them be more efficient, safe and organised. Despite being confronted with difficulties, participants also felt that minimal assistance reduced their stress and increased their confidence in their own abilities. They finally appreciated the idea that minimal cognitive assistance could be provided by informal caregivers or ATCs, although some expressed doubts about their ability to offer such personalised assistance.

This study highlighted the relevance and acceptability of minimal cognitive assistance from the point of view of participants, as it gave them the opportunity to do most of the task by themselves, while receiving the support required to progress. By promoting independence and self‐confidence, this approach is consistent with positive supports and strength‐based approaches such as apprenticeship, which are perceived as more collaborative and less confrontational than other performance‐based approaches (Hammell, 2016; Ylvisaker et al., 2003). In line with evidence‐based rehabilitation interventions such as error‐based learning (Ownsworth et al., 2017) and the guided discovery component of the CO‐OP intervention (Dawson et al., 2013), two approaches used by occupational therapists to guide the person in identifying solutions when confronted with difficulties, minimal cognitive assistance could promote the use of residual abilities and success in complex everyday activities while supporting the person's cognition. These similarities suggest that if used regularly, minimal cognitive assistance could have therapeutic potential in integrating skills and optimising independence in complex everyday activities, despite being provided outside a rehabilitation setting. As depicted during the therapist‐guided sessions, the adaptive approach of minimal cognitive assistance promotes personalised assistance based on the person's needs, therefore providing guidance in steps previously identified as challenging, while progressing assistance only when necessary. Such an approach is similar to the vanishing cues methods (Glisky, 2011), in which the therapist combined principles of backward and forward chaining to allow the person to progress in the task while reducing support given over time and practice. Considering how TBI is a dynamic condition (Corrigan et al., 2025), minimal cognitive assistance could be provided regularly to promote engagement in meaningful activities and empower people with TBI in identifying and building on their own strengths, including via ATCs.

However, providing personalised assistance may be challenging. Although participants appreciated the idea of receiving minimal cognitive assistance from formal and informal caregivers or by ATCs, providing such assistance requires a thorough evaluation and monitoring, as well as finding a balance between letting the person do things by themselves (which may cause frustrations when confronted with difficulties) and support (which may feel like an intrusion, as mentioned by Anna). Consequently, although the person should be provided opportunities to try things out before receiving assistance, special attention should be given to their emotional well‐being (Gagnon‐Roy et al., 2021). These issues highlight the need for both formal and informal caregivers to be trained in providing minimal cognitive assistance, potentially by occupational therapists, as they must continuously adapt their approach to the person's needs, actions, difficulties and emotional responses (Gagnon‐Roy et al., 2021). For formal caregivers, integrating minimal cognitive assistance in collaboratively selected meaningful activities is especially relevant for quality of care, in line with a person‐centred approach (Topping et al., 2022). Principles from communication partner training, an evidence‐based approach recognised as improving communication following TBI (Togher et al., 2023), may be used to support caregivers in providing minimal cognitive assistance in a positive manner. For example, skill‐building and problem‐solving techniques could be used to help caregivers give pauses to the person with TBI and ask positive, nondemanding questions, in line with positive communication (O'Rourke et al., 2018; Togher et al., 2004). Promoting collaboration and communication between caregivers and the person with TBI could also help define types of assistance that are acceptable within their relationship.

Although participants were interested in using ATCs, they also mentioned some doubts about the technology's ability to provide personalised assistance. Advancements in activity recognition and artificial intelligence are, however, promising, as they could support precise monitoring and adaptive assistance based on the person's actions (Doukaga et al., 2025; Olivares et al., 2016; Pomare et al., 2024; Tekemetieu et al., 2021, 2023). Initial ATCs' configuration and continuous adaptations could also help in ensuring the best match between the assistance offered and the individual's needs (Gagnon‐Roy et al., 2025; Gilles et al., 2025). Contextual factors, such as available resources and healthcare workflow rigidity, should, however, be taken into account when integrating ATCs (Petsani et al., 2025). Further studies are needed to develop and better configure ATCs so that they could provide personalised and minimal assistance in an acceptable manner to adults with TBI.

Using a multistep approach involving an observation‐based evaluation using the IADL Profile and a guided session, this qualitative descriptive multiple case study provided a detailed understanding of the perceptions of five individuals with a TBI and presenting diverse characteristics, difficulties and needs. Beyond documenting the minimal cognitive assistance provided to participants and how they perceived such assistance, our study included video feedback as an innovative step that could potentially optimise the acceptability of ATCs and minimal cognitive assistance. In addition to helping improve self‐awareness regarding experienced difficulties and needs (Schmidt et al., 2013), the use of videoclips of previous performances allowed participants to better comprehend the assistance's objectives and their own reactions, thus helping both the participants and the occupational therapists in recognising optimal assistance to provide in the future. Integrating a video feedback session when configuring ATCs could thus help better match the needs of people with TBI, their perceptions of their own performance and the ATC configuration.

Although using a rigorous methodology, this study also had some limitations. In addition to not using a validated guide, the interviews exploring the perceptions of participants regarding minimal cognitive assistance were not conducted immediately following the intervention. This may have reduced the participants' ability to remember how they felt about the given assistance. However, the use of videos supported them in recalling their performance. Moreover, their perceptions were based on only two sessions and may not represent how they would feel if minimal cognitive assistance was provided daily over a prolonged period of time, highlighting the need for future studies. Participants also did not check emerging themes, although they were validated by experts in TBI. As assistance was provided by a person, results may also vary from the experience of participants if assistance was provided via ATCs. However, this manner of providing assistance allowed for more personalisation and flexibility, without technical and feasibility constraints. Finally, only five participants were included in the multiple case study, thus reducing the generalisability of our results. We were also not able to attain data saturation due to the heterogeneity of the sample. Nonetheless, each case was extensively described, including their context, difficulties and assistance, to improve our results' credibility and transferability.

## 5 CONCLUSIONS

This qualitative descriptive multiple case study aimed to document the perceptions of individuals with TBI when provided minimal cognitive assistance by occupational therapists, as well as the acceptability of potentially having a caregiver or ATC providing personalised assistance in their everyday activities. Based on participants' perceptions, minimal cognitive assistance is relevant as it was helpful in planning tasks and completing them efficiently, safely and satisfactorily while giving them the time and space to use their residual abilities and strengths. Although participants required more time and energy to complete the tasks than if instructed step‐by‐step, they felt more independent and confident in their own abilities. Although minimal cognitive assistance could be provided by caregivers and ATCs, further studies are required to better support family members or paid support staff in providing personalised assistance and developing technologies that are acceptable and adaptable to individuals with a TBI. Moreover, while the present study captured the perception of participants with TBI during a personalised process, future studies should focus on their perceptions when assistance is provided during daily activities within their home environment, on both good days and bad days. Such understanding could support further adjustments of cognitive assistance to optimise the person's performance in meaningful activities and support future ATCs development.

## AUTHOR CONTRIBUTIONS

The authors declare that they all have contributed significantly and that they are all in agreement with the content of the manuscript. **Mireille Gagnon‐Roy:** conceptualisation, investigation, visualisation, writing—original draft preparation (major). **Nathalie Bier:** conceptualisation, funding acquisition, validation, writing—review and editing. **Frédérique Poncet:** investigation, validation, writing—review and editing. **Leanne Togher:** validation, writing—review and editing. **Mélanie Amaral Dos Santos:** formal analysis, visualisation, writing—original draft preparation. **Carolina Bottari:** conceptualisation, funding acquisition, supervision, validation, writing—review and editing.

## CONFLICT OF INTEREST STATEMENT

The authors declare no conflicts of interest.

## DECLARATION OF USE OF ARTIFICIAL INTELLIGENCE

Certain sentences were entered into Co‐Pilot (TM) with the prompt to review for English. The plain language summary was also entered with the prompt to rewrite to a Flesch–Kincaid reading level no greater than 8″. Outputs were checked and confirmed by the authors before inclusion.

## Supporting information


**Data S1** Supporting Information.

## Data Availability

The data that support the findings of this study are available on request from the corresponding author. The data are not publicly available due to privacy or ethical restrictions.
