# Supplementary material for: How does it feel to be helped using minimal cognitive assistance following a traumatic brain injury?
Source: Aust Occup Ther J. 2026 Jul 9;73(4):e70102. doi: 10.1111/1440-1630.70102 (PMC13348017; doi:10.1111/1440-1630.70102)
Supplement: Supplementary file 1 — Data S1 Supporting Information. [file AOT-73-0-s001.docx]

| ***Supplementary file: Description of the selected moments of assistance presented during the individual interviews*** | |
| --- | --- |
| ***Participants*** | ***Selected moments of assistance*** |
| ***James*** | ***From the IADL Profile***  *Grocery shopping task*:   1. Clarification provided by the therapist to help James understand the context and formulate the goal of grocery shopping: “*We would like to see you function outside of the kitchen. You proposed a meal with what you had in your fridge. What would you do if you had nothing?*”   *Budgeting task:*   1. Assistance questioning James to help him think about expenses he forgot: “*You plan on putting $13,080 (of the $25,000 annual amount) on the car?”*   ***From the therapist-guided session***   1. Assistance questioning James about the recipe to help him read it through: “*Do you think that you read all the steps required to prepare a lasagna?”* 2. Assistance to stimulate thought processes and plan the next steps: “*Could you tell me how you will do it?”* 3. Assistance to stimulate thought processes and find strategies to be more efficient: “*But boiling water can take some time. What could you do in the meantime?”* 4. Assistance to further stimulate thought processes as James mentions he will put the pasta in the boiling water: “*Are you sure? What could you do during the time water is boiling?”* 5. Cue to obtain a more successful result as James forgot an important step: “*Maybe you could wash your hands with soap before starting?”* 6. Assistance to stimulate thought processes and challenge James to help better organise his working space: “*What could you do to be better organise? Do you need to put this casserole here (which will be used only in the last steps)?* 7. Cues to help James obtain a better result and be more efficient: *“Onions should be smaller. Go!”* 8. After James stopped to wonder what the next step is, and propose an inadequate one, assistance to challenge him and think about the next step: “*Check. Chopped onion? Is there another step you need to do?”* 9. After James had shredded the cheese, assistance aiming to help him be more efficient: “*What is the next step? What do you have to do?* 10. Cues aiming to help James follow the steps to assemble the lasagna (via modelling) |
| ***Mary*** | ***From the IADL Profile***  *Meal preparation task*:   1. Interaction aiming to encourage independence: “*You organise yourself like you want to.”* 2. Cue to help Mary find the recipe she had in mind: *“Do you remember on what website it was?”* 3. Assistance to question Mary (i.e., challenging) as she begins to prepare the pasta before placing her meatloaf in the oven: “*Now, just to make sure, what are we doing?”* 4. Feeback on Mary performance during the meal preparation task to highlight her strengths and improve her self-confidence   *Budgeting task:*   1. Step-by-step assistance to help Mary complete the task as she is mentioning not being able to do it.   ***From the therapist-guided session***   1. Cue to make sure everything is available before beginning the recipe: “*Before starting the recipe, could we make sure we have everything we need?”* 2. Assistance to stimulate Mary thought processes and help her plan the task: “*How will you organise yourself? What will you do first?*” 3. Assistance to help Mary monitor the task and identify her mistakes (did not put a timer): “*Before preparing the icing, how will we know the cakes are ready?*” 4. Cue to improve performance and obtain a better result: “*You can stop mixing the butter before it turns into cheese.”* 5. Assistance to challenge Mary and help her identify her mistake: “*Is it written in the recipe?*” |
| **Anna** | ***From the IADL Profile***  *Meal preparation task*:   1. Assistance to stimulate Anna thought processes to identify a meal to prepare: “*Since you are not at home, is there another way to find an idea?*” 2. Interaction to encourage Anna’s independence when making a choice: “*It’s your choice.”* 3. Assistance to question Anna (i.e., challenging) to help her plan the meal before starting it: “*Before boiling water, could you remind me what meal you are preparing?*” 4. Cues to follow the steps so that the pasta and vegetables are well-cooked: “*What time for the vegetables? If we just add the pasta in the water and that is approximately the same time, what are we doing with the vegetables?”*   *Obtaining an information task:*   1. Assistance to stimulate though processes and help her find alternatives: *“Is there other places you could find this info? If you were at your home?*”   ***From the therapist-guided session***   1. Assistance to stimulate thought processes and help plan the task: “*If we are going back to the recipe, could you tell me what you are planning to do?*” 2. Assistance to challenge Anna before starting the recipe: “*Before starting, do we have everything we need?”* 3. Assistance to help further plan and initiate the task: *“Now, what are we doing?*” 4. Assistance to challenge Anna about the steps order: “*Are you ready to add the eggs? What should you do before? Did you check the ingredients?”* 5. Cues and physical assistance to help efficiently cut vegetables 6. Assistance to help stimulate though processes and initiate the next step: “*What are we doing with this bowl?*” |
| **Sarah** | ***From the IADL Profile***  *Meal preparation task*:   1. Assistance to remind Sarah and challenge her about the evaluation context: “*Before continuing, do you remember the $20? If I told you that I need you to use it…”* 2. Assistance to help Sarah find ideas when confronted with a difficulty: “*I see other options…”* 3. Cue to help Sarah be more efficient when following the steps: “*You could do that later, and maybe begin boiling water. Because it will take some time to cut all your vegetables.”* 4. Assistance to question Sarah to help her follow the recipe: “*Was it before or after the zucchinis? You mention putting them aside.”* 5. Assistance to help Sarah initiate a step: “You add the onions?” 6. Assistance to help Sarah think about what she could do while waiting: “*Since you must wait some time, what could you do in the meantime? That is not necessarily related to your recipe?”* 7. Physical assistance to help transfer the sauce with the pasta.   *Obtaining an information task:*   1. Assistance to scaffold on ideas previously mentioned by Sarah: “*You mention multiple possibilities. Call a phone number, using the phone book, or Internet.”* 2. Assistance to challenge Sarah about the objective of the task: “*What was the mode of transportation I mentioned?*” 3. Step-by-step assistance to select a website: “*You already tried this one, it does not work. Could you try this one?”*   ***From the therapist-guided session***   1. Assistance to help Sarah plan the task by stimulating her thought processes: “*Now that you selected your recipe, what will you do?”* 2. Cues to help select and taking out the good tools for the recipe (e.g., bowls, cutting board) 3. Assistance to reactivate Sarah and help her initiate the next step: “*In the second bowl, what do you need to add?”* 4. Physical assistance to demonstrate how to cut herbs. |
| **Brian** | ***From the IADL Profile***  *Meal preparation task*:   1. Assistance to restart Brian’s thought processes to help him select a meal: “*Finally, what will you do? You have a lot of ideas.”* 2. Assistance to remind and scaffold on previous ideas of Brian to help him make a choice: *“Could you tell me what you want to do? You mentioned a desert?”* 3. Feedback on Brian’s performance and some of his mistakes: *“How would you evaluate yourself on this activity?”*   *Obtaining an information task:*   1. Assistance to challenge and think about alternate ways of obtaining an information: *“Do you think there could be another way to obtain this information besides calling or going in person?”*   ***From the therapist-guided session***   1. Assistance to stimulate thought processes and find an idea of meal: *“You told us you have difficulty finding new ideas of meals. How could you find another idea?”* 2. Cue to initiate the search of a new recipe: *“If I name you a website, would you be open to search on this one?”* 3. Assistance to stimulate thought processes and find alternatives: *“What would you like to prepare?”* 4. Cue to help find an alternative and adequately adapt the recipe: *“You could replace by a can of tuna, some ham, or something else…*” 5. Assistance to challenge Brian and remind him of previous difficulties: *“Do you remember what happen with the quantities last time? I am not sure you have enough water.”* 6. Assistance to challenge and cue Brian about a potential safety issue: *“You added oil, but you did not peel the onion?”* 7. Cue and physical assistance to obtain a better result: *“Shouldn't they be cut? Would it be okay if I cut them in half to ensure the pasta cooks properly”* 8. Cue to help Brian be more efficient and initiate another task while waiting: “*Maybe you could set the table while waiting for your meal.*” |
